# Dehydration in the nursing home: Recognition and interventions taken by Dutch nursing staff

**DOI:** 10.1111/jan.15032

**Published:** 2021-08-30

**Authors:** Simone J. C. Paulis, Irma H. J. Everink, Ruud J. G. Halfens, Christa Lohrmann, Jos M. G. A. Schols

**Affiliations:** ^1^ Department of Health Services Research Care Public Health Research Institute (CAPHRI) Maastricht University Maastricht The Netherlands; ^2^ Institute of Nursing Science Medical University of Graz Graz Austria; ^3^ Department of Family Medicine Care Public Health Research Institute (CAPHRI) Maastricht University Maastricht The Netherlands

**Keywords:** certified nurse assistants, dehydration, diagnostic strategy, intervention, knowledge, nursing home, registered nurses

## Abstract

**Aims:**

To examine which signs/symptoms registered nurses (RNs) and certified nurse assistants (CNAs) (nursing staff) in Dutch nursing homes associate with dehydration, if they observe these signs/symptoms themselves and what they do after observing them.

**Design:**

A cross‐sectional study.

**Methods:**

In February 2020, using an online questionnaire based on a diagnostic strategy to diagnose dehydration, nursing staff was asked: (1) which signs/symptoms they associate with dehydration; (2) if they observe these signs/symptoms themselves; and (3) which actions they take after observing these signs/symptoms in a resident. Descriptive statistics and Chi‐square statistics were used to describe the answers and explore significant differences between groups.

**Results:**

In total, 250 RNs and 226 CNAs participated. Among RNs, 67%–99% associated the signs/symptoms of the strategy to dehydration compared with 45%–98% of the CNAs. RNs and CNAs often indicated to observe signs/symptoms from the strategy themselves (80.1% and 92.6%), but they also often relied on information given by other care professionals and the informal caregiver. Interventions taken were mainly focused on communicating findings to colleagues.

**Conclusion:**

Many signs/symptoms from the diagnostic strategy trigger nursing staff to think of dehydration. Results also show that a variety of formal and informal caregivers are involved in dehydration care. As RNs and CNAs did often not receive dehydration training after entering workforce, this could have limited their ability to recognize signs/symptoms related to dehydration. To ensure timely recognition of dehydration, a clear description of roles and responsibilities about dehydration care in, and between, formal and informal caregivers is essential with structurally embedded dehydration training in the nursing home.

**Impact:**

Tackling dehydration in the nursing home requires interdisciplinary collaboration and communication with family members. Without clear roles and responsibilities, a risk of dehydration can be left unattended.

## 1 INTRODUCTION

When the human body does not receive the right amount of water it needs to function, a state of dehydration may occur (Anjo et al., 2020). Dehydration is associated with negative outcomes such as falls, delirium, kidney failure (Lešnik et al., 2017) and increased mortality risk (Bunn & Hooper, 2019).

**FIGURE 1:**
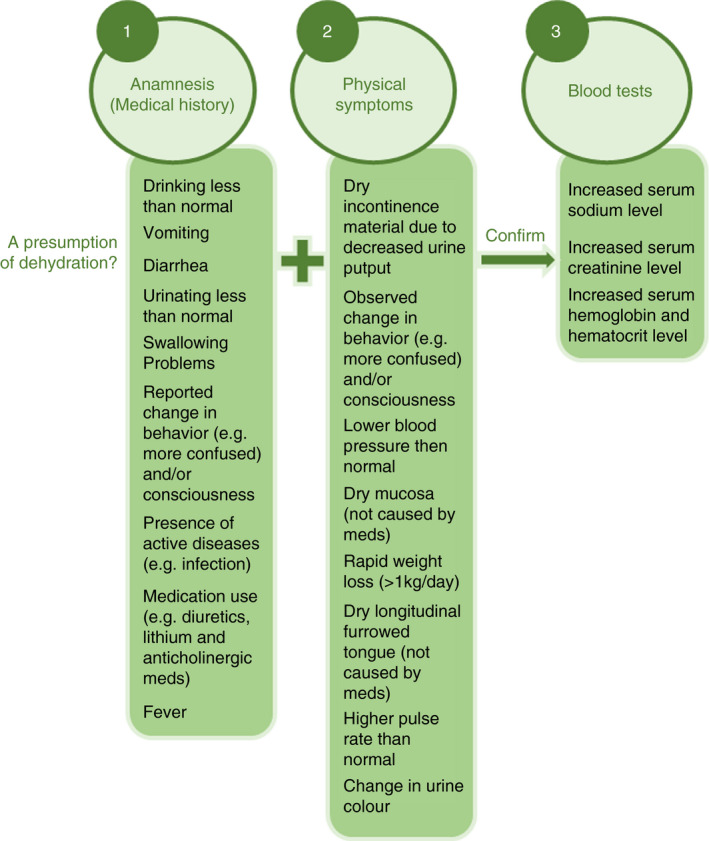
Diagnostic strategy to diagnose dehydration in the nursing home

Dehydration is common among nursing home residents (residents) all over the world (Lešnik et al., 2017), where international prevalence rates up to 38.5% are found (Paulis et al., 2018). To diagnose dehydration in residents, various methods are described in the literature, including checking physical symptoms, blood tests and urine tests. However, each method has its own limitations, such as lack of diagnostic accuracy in this specific population (e.g. dry mucosa due to use of specific medication) or accessibility of tests in the nursing home (e.g. blood tests) (Hooper et al., 2014). To assess which method to diagnose dehydration is both relevant and feasible to use in the nursing home, Paulis et al. (2020) performed a Delphi study to reach international consensus on this topic. This resulted in a diagnostic strategy to diagnose dehydration consisting of a presumption phase and a confirmation phase (see Figure 1). In the presumption phase, care professionals have to check the presence of various anamnestic items related to dehydration (e.g. drinking less than normal or presence of active diseases), as well as various physical symptoms related to dehydration (e.g. dry longitudinal furrowed tongue or rapid weight loss). The clinical view of the caregiver, which is not only taking these signs and symptoms into account but also characteristics of the individual resident (e.g. co‐morbidity that could influence these symptoms) and the resident's care environment, is the leading source in deciding whether or not the confirmation phase will be performed. In this phase, blood tests (testing serum sodium, serum creatinine, serum haemoglobin and serum haematocrit) will be executed to check if a resident actually suffers from dehydration.

This study examines which of the signs and symptoms of the presumption phase from the diagnostic strategy are associated with dehydration by registered nurses (RNs) and certified nurse assistants (CNAs) in Dutch nursing homes. In addition, this study also investigates whether RNs and CNAs actually observe these signs and symptoms in daily practice themselves and what action(s) they take after observing these signs and symptoms.

### 1.1 Background

Dutch nursing homes employ a variety of caregivers, such as nursing home physicians (NHPs ‐ physician specialized in the care of frail residents with chronic, complex diseases), advanced nurse practitioners (ANPs) and nursing staff (e.g. RNs, CNAs, nurse assistants [NAs], nutritional assistants and allied health professionals) (Backhaus, 2017; Kuk et al., 2018; Verenso, the Dutch Association of Elderly Care Physicians, 2015). Furthermore, informal caregivers often have a supporting active role in the nursing home as well (Roberts & Ishler, 2018). To assess if nursing home residents have (a risk of) dehydration using the internationally agreed on diagnostic strategy, nursing home professionals require knowledge about dehydration, skills to observe dehydration (risks) and frequently interact with the resident. It is expected that mostly nursing staff (RNs and CNAs) most frequently interact with the resident, and therefore have an important role in applying the presumption phase of the diagnostic strategy (Kuk et al., 2018; see Figure 1). Applying this phase requires knowledge about which signs and symptoms are related to dehydration, as well as up‐to‐date knowledge about the health status of the resident and an ability to observe changes in it. If these skills are lacking, changes of the residents’ clinical status will not be detected or associated with dehydration. As a consequence, dehydration might not be recognized in time (Pickenhan et al., 2020). However, some issues with regard to knowledge about dehydration and skills to perform dehydration care in the nursing home should be mentioned. First, various sources state that knowledge of nursing staff about dehydration seems insufficient in nursing homes and there seems to be a difference in knowledge base between the different educational levels of nursing staff (Beattie et al., 2012; Oates & Price, 2017; Pickenhan et al., 2020). For example, Bauer et al. (2015) stated that signs and symptoms of dehydration were significantly more often acknowledged by RNs than by lower educated nursing staff, such as CNAs. This is a potential problem, as lower educated nursing staff levels (e.g. CNA) are often responsible for the majority of direct resident care, making them the first to report deterioration of clinical status and adverse events (Beattie et al., 2012). Moreover, even if nursing staff (e.g. RNs and CNAs) observes dehydration signs and symptoms in a resident, there is no literature available on actions they take next. Therefore, it is important to know more about which signs and symptoms of the dehydration strategy are associated with dehydration and observed by nursing staff, and which actions nursing staff takes after having observed potential dehydration risks.

## 2 THE STUDY

### 2.1 Aims

The aim of this study is threefold: (1) to examine which signs and symptoms RNs and CNAs in Dutch nursing homes associate with dehydration; (2) to examine if RNs and CNAs notices these signs and symptoms in daily practice themselves or if they receive this information from others; and (3) to assess what RNs and CNAs actually do after observing the presence of these signs and symptoms in a resident.

### 2.2 Study design

The design of this study is cross‐sectional. An online questionnaire was developed and disseminated among baccalaureate‐educated or vocationally trained RNs and CNAs.

### 2.3 Participants

Professionals eligible to participate in this study were RNs and CNAs.

To be included in this study, professionals:

- were currently working in a nursing home in the Netherlands;
- agreed on the use of their answers for research purposes

Participants were recruited in February 2020 through the Dutch association for RNs and CNAs (V&VN), educational institutes for RNs and CNAs and through a convenience sample of nursing homes in the Netherlands. The association, educational institutes and nursing homes received an e‐mail from the first author, asking to disseminate the questionnaire to their (former) students, members and employees. Also, instructions to complete the questionnaire using the online Qualtrics software (University of Michigan‐Flint, 2020) were provided. Participation was voluntarily and anonymous and the questionnaire link could only be used once. A reminder was sent to the association, educational institutes and nursing homes in June 2020.

### 2.4 Data collection

#### 2.4.1 Questionnaire

As there is no instrument available to assess the study aims, a questionnaire was developed. The basis of this questionnaire was a diagnostic strategy to diagnose dehydration, designed by Paulis et al. (2020).

The first part of the questionnaire focused on the background characteristics of participants. Questions about profession (RN or CNA), years of working experience (0–5 years, 5–10 years, 10–15 years, 15–20 years or >20 years) and whether or not the professionals received training on dehydration during and after their education (yes or no) were included. In addition, participants were asked to answer if a protocol/guideline for diagnosing and treating dehydration was available in the nursing home they worked in and which minimum amount of fluid per day per resident was recommended.

In the second part of the questionnaire, professionals were first asked to describe which signs and symptoms trigger them to think of dehydration in residents, using an open text field.

Thereafter, professionals received a list of all signs and symptoms associated with dehydration and based on the presumption phase of the diagnostic strategy. Professionals were asked to indicate:

1. To what extent they associate these specific signs and symptoms with the presence of dehydration in a resident. Answer options were ‘always’, ‘sometimes’ and ‘never’.
2. If the participant is usually the first one to notice these signs and symptoms when interacting with a resident, or if they receive this information from someone else. Answer options were ‘I notice this myself’ or ‘someone else notices this and informs me’. If the answer ‘someone else notices this and informs me’ was chosen, the participant was asked to indicate who observed and provided the information to them (NHP, ANP, RN, CNA, NA or the informal caregiver). Multiple answers could be chosen for this question.
3. Which actions the participant takes after observing the signs and symptoms associated with dehydration. For each sign/symptom (see Figure 1), various answering options were formulated based on available literature and clinical experiences of some research team members who are working in Dutch nursing homes themselves (Malarvizhi & Gugan, 2019). The full questionnaire can be found in Supporting Information Additional file 1. An example of answer options for the sign ‘vomiting’ was: (a) I write it down in the agenda for the NHP/ANP visit; (b) I communicate this to the NHP or ANP; or to the (c) RN; (d) care coordinator; (e) CNA; (f) NA; (g) I start a drink record chart; (h) I give the resident more fluid; (i) I give extra fluid through a hypodermoclysis; (j) I perform additional physical examinations; (k) I request blood tests; (l) I don't do anything; (m) other. Again, multiple answers could be chosen. For the items ‘drinking less than normal’ an extra answer option ‘contacting the dietician’ was added. For the item ‘swallowing problems’ participants could choose the answer option ‘contacting the speech therapist’.

### 2.5 Ethical considerations

The study was approved by the local Medical Ethics Committee of a University Hospital (2019–1443).

To guarantee data security the collected data are stored on the secure server of the corresponding university. In addition, to secure anonymity of the participants, private information such as the name of the participant and the organization he/she worked was not requested so that the participant could not be traced.

### 2.6 Data analysis

The results of the questionnaire were analysed using descriptive statistics in SPSS statistics 26 IBM (George & Mallery, 2019). Differences between RNs and CNAs about baseline characteristics are calculated using the Chi‐square statistics. Chi‐square statistics were also used to examine significant differences between RNs and CNAs in associating signs and symptoms of the diagnostic strategy with dehydration (answer categories ‘never’ and ‘always or sometimes’) in residents, who observes signs and symptoms of dehydration (RNs and CNAs their self) and to explore differences between RNs and CNAs about interventions taken when dehydration signs and symptoms were present in a resident. The significance level was set at <0.01 (George & Mallery, 2019).

### 2.7 Validity, reliability and rigour

To ensure the quality of the questionnaire, a test panel (*n* = 12) was involved to assess the questionnaire on content and clarity. The panel consisted of NHPs (*n* = 3), ANPs (*n* = 2), RNs (*n* = 2), CNAs (*n* = 3) and NAs (*n* = 2). Respondents of the test panel were obtained through convenience sampling, using national contacts from the first author. The test panel received the pilot questionnaire by e‐mail or received a printed version. We asked the respondents of the test panel to critically reflect on the questionnaire by assessing (1) if the questions corresponded with the purpose of the study; (2) if the wording of the questions was clear and understandable; and (3) if any substantive information was missing. The comments of the test panel could be returned in writing or by e‐mail to the first author. After critical revision by the test panel, the questionnaire was adjusted (by adding some textual changes) and finalized (see Supporting Information Additional file 1).

## 3 RESULTS

### 3.1 Background characteristics

In total, 476 questionnaires were completed. The questionnaire was filled in by 250 (52.5%) RNs and 226 CNAs (47.5%). The majority of the professionals had >20 years (35.9%) of working experience in the nursing home, and 41.6% of all participants worked with psychogeriatric residents (Table 1).

**TABLE 1:** Baseline characteristics participants

	CNA (*n* = 226)^a^	RN (*n* = 250)^b^	Total (*n* = 476)	*p*‐value*
Working experience (years)				
0–5	18.6%	29.6%	24.4%	0.001
5–10	11.1%	17.6%	14.5%	
10–15	15.5%	13.2%	14.3%	
15–20	10.2%	11.6%	10.9%	
>20	44.7%	28.0%	35.9%	
Category residents				
Somatic	20.4%	25.6%	23.1%	<0.001
Psychogeriatric	52.2%	32.0%	41.6%	
Both	27.4%	42.4%	35.3%	
Received dehydration training during education				
Yes	71.2%	85.2%	78.6%	<0.001
Received dehydration training after education				
Yes	24.8%	19.2%	21.8%	0.141
Protocol/guideline dehydration care available in nursing home				
Yes	47.3%	37.6%	42.2%	<0.001
No	7.1%	24.8%	16.4%	
Don't know	45.6%	37.6%	41.4%	

^a^ CNA = Certified nurse assistant.

^b^ RN = Registered nurse.

* *p*‐value is calculated using chi‐square statistics and *α* = 0.01. Compares baseline characteristics between CNAs and RNs.

To the question whether or not the professional received training about dehydration care during their education, 78.6% (*n* = 374) answered with ‘yes’. After entering the workforce, 21.8% (*n* = 104) received additional (post‐vocational) training on dehydration. Of the 476 professionals who completed the questionnaire, 107 (47.3%) CNAs and 94 (37.6%) RNs were aware of the presence of a protocol/guideline on dehydration care in the nursing home they were working at (see Table 1). Finally, the median recommended daily fluid intake for residents in the nursing home indicated by participants was 1,400 ml. Significant differences between CNAs and RNs about the baseline characteristics were found for working experience (*p* = 0.001), category residents (*p *< 0.001), dehydration training during education (*p *< 0.001) and the presence of a protocol/guideline on dehydration care in the nursing home they worked at (*p *< 0.001).

### 3.2 Signs and symptoms associated with dehydration

To the initial open text question ‘Which signs and symptoms trigger you to think of dehydration in residents?’, the answers ‘confusion’ (31.9%), ‘change in urine colour’ (29.6%), ‘urinating less than normal’ (29.0%), ‘being drowsy’ (28.4%) and ‘decreased skin turgor’ (25.2%) were mostly written down by the participants (>25%).

After asking this question again using the pre‐structured list of signs and symptoms, ‘drinking less than normal’, ‘diarrhea’, ‘urinating less than normal’, ‘medication use related to dehydration’, ‘fever’, ‘dry mucosa’, ‘change in urine colour’ and ‘dry incontinence material’ were indicated by >90% of the RNs and CNAs as signs that ‘always or sometimes’ made them think of dehydration. The signs and symptoms that made RNs and CNAs most often (>20%) ‘never’ think of dehydration were ‘higher pulse rate than normal and ‘lower blood pressure than normal’. These results are also shown in Table 2.

**TABLE 2:** The extent to which signs and symptoms of the diagnostic strategy make RNs and CNAs think of dehydration

	CNA (*n* = 226)^a^		RN (*n* = 250)^b^		*p*‐value*
	Never (%)	Always or sometimes (%)^c^	Never (%)	Always or sometimes (%)^c^	
Drinking less than normal	2.2%	97.8%	0.8%	99.2%	0.201
Vomiting	11.1%	88.9%	1.2%	98.8%	<0.001
Diarrhoea	4.0%	96.0%	1.6%	98.4%	0.111
Urinating less than normal	4.0%	96.0%	1.6%	98.4%	0.111
Medication use related to dehydration	8.4%	91.6%	3.6%	96.4%	0.026
Presence of active disease(s)	14.6%	85.4%	6.4%	93.6%	0.003
Change in behaviour	17.7%	82.3%	12.0%	88.0%	0.080
Swallowing problems	19.9%	80.1%	9.2%	90.8%	0.001
Fever	3.5%	96.5%	2.8%	97.2%	0.645
Lower blood pressure than normal	46.5%	53.5%	24.0%	76.0%	<0.001
Dry mucosa	9.7%	90.3%	1.6%	98.4%	<0.001
Rapid weight loss	25.2%	74.8%	18.0%	82.0%	0.055
Dry longitudinal furrowed tongue	19.0%	81.0%	16.0%	84.0%	0.385
Higher pulse rate than normal	54.9%	45.1%	33.2%	66.8%	<0.001
Change in urine colour	3.5%	96.5%	0.8%	99.2%	0.037
Dry incontinence material	3.1%	96.9%	2.8%	97.2%	0.848

^a^ CNA = Certified nurse assistant.

^b^ RN = Registered nurse.

^c^ Answer categories ‘always’ and ‘sometimes’ are combined.

* *p*‐value is calculated using chi‐square statistics and *α* = 0.01. Compares answer categories ‘never’ and ‘always or sometimes’ provided by CNAs versus RNs.

If we look at the differences in answers provided by CNAs and RNs, it appeared that CNAs significantly more often indicated to ‘never’ associate the following signs and symptoms with dehydration compared with RNs: ‘vomiting’, ‘presence of active diseases’, ‘swallowing problems’, ‘lower blood pressure than normal’, ‘dry mucosa’ and ‘higher pulse rate than normal’ (see Table 2).

If we relate these results to the question whether or not professionals received any form of education on the topic of dehydration after entering the workforce, CNAs and RNs who answered with ‘always or sometimes’ on the question ‘are you triggered to think of dehydration when a resident exhibits this sign/symptom?’ seemed to have received more education (range among CNAs 50.0%–98.2% and range among RNs 72.9%–100.0%) compared with the group who answered this question with ‘never’ (range among CNAs 1.8%–50.0% and among RNs 0%–27.1%).

### 3.3 Observing signs and symptoms associated with dehydration

When looking at the question which care professional is observing the signs and symptoms related to dehydration, on average 80.1% of the RNs and 92.6% of the CNAs indicated to observe the different signs and symptoms themselves (see Table 3). For almost all items, there was a statistically significant difference (*p *< 0.01) except for ‘medication use related to dehydration’, ‘lower blood pressure than normal’ and ‘higher pulse rate than normal’, where no differences between CNAs and RNs were observed.

**TABLE 3:** Observed signs and symptoms of the diagnostic strategy by CNAs and RNs themselves

	CNA (*n* = 226)^a^	RN (*n* = 250)^b^	*p*‐value*
Drinking less than normal	96.0%	80.8%	<0.001
Vomiting	96.5%	81.6%	<0.001
Diarrhoea	98.2%	80.4%	<0.001
Urinating less than normal	96.0%	78.0%	<0.001
Medication use related to dehydration	85.4%	83.2%	0.511
Presence of active disease(s)	92.0%	82.8%	0.003
Change in behaviour	95.6%	81.6%	<0.001
Swallowing problems	93.8%	77.2%	<0.001
Fever	96.0%	86.4%	<0.001
Lower blood pressure than normal	85.8%	76.8%	0.012
Dry mucosa	94.7%	82.0%	<0.001
Rapid weight loss	87.2%	76.4%	0.003
Dry longitudinal furrowed tongue	87.2%	75.2%	0.001
Higher pulse rate than normal	84.5%	78.8%	0.109
Change in urine colour	96.0%	83.6%	<0.001
Dry incontinence material	96.5%	78.0%	<0.001
	Mean 92.6%	Mean 80.1%	

^a^ CNA = Certified nurse assistant.

^b^ RN = Registered nurse.

* *p*‐value is calculated using chi‐square statistics and *α* = 0.01. Compares answer categories ‘observe myself’ provided by CNAs versus RNs.

If participants (CNAs and RNs) indicated to receive information about the signs and symptoms from someone else, they most often received this information from a colleague CNA (on average for all symptoms 78.3% for RNs and 59.0% for CNAs), followed by receiving information from NAs (on average for all symptoms 51.9% for RNs and 48.2% for CNAs). Another frequently mentioned person receiving information from about the signs and symptoms was the informal caregiver (29.5% for RNs and 28.0% for CNAs). This person was an important information source for information about ‘drinking less than normal’, ‘vomiting’, ‘change in behaviour’, ‘swallowing problems’ and the presence of diarrhoea. Lastly, for information about ‘medication use related to dehydration’, the NHP/ANP appeared to be an important information source for RNs and CNAs. All answers given to this question can be found in Supporting Information Additional file 2.

### 3.4 Interventions to treat dehydration

Table 4 describes which interventions per professional (RNs and CNAs) are applied after being aware of the presence of signs and symptoms associated with dehydration. The most frequently (>30%) mentioned interventions for RNs and CNAs were: (1) I communicate this verbally to the NHP/ANP or write it down in the agenda for the NHP/ANP visit, (2) I give the resident more fluid and (3) I communicate this to a (colleague) CNA.

**TABLE 4:** Interventions taken by CNAs and RNs after observing signs and symptoms from the diagnostic strategy

Intervention	CNA (*n* = 226)^a^^,^ e	RN (*n* = 250)^b^^,^ e	*p*‐value*
I communicate this verbally with the NHP/ANP or write it down in the agenda for the NHP/ANP visit^c^^,^ f	76.5%	81.7%	0.146
I give the resident more fluid	51.3%	55.6%	0.169
I communicate this to a (colleague) RN^b^	46.8%	28.5%	<0.001
I communicate this to a (colleague) CNA^a^	41.5%	36.2%	0.139
I communicate this to the care coordinator	33.8%	28.0%	0.581
I start a drink record chart	28.5%	45.9%	<0.001
I communicate this to a (colleague) NA^d^	27.7%	25.6%	0.112
I perform additional physical examinations	13.0%	26.0%	0.001
I request blood tests	3.4%	3.1%	0.570
I give extra fluid through a hypodermoclysis	1.5%	5.5%	0.004
I don't do anything	1.4%	2.0%	0.154

^a^ CNA = Certified nurse assistant.

^b^ RN = Registered nurse.

^c^ NHP/ANP = Nursing home physician/advanced nurse practitioner.

^d^ NA = Nurse assistant.

^e^ Percentage calculated on the total answers per intervention for 16 signs/symptoms of the diagnostic strategy.

^f^ Answer options ‘I write it down in the agenda for the NHP/ANP visit’ and ‘I communicate this with the NHP/ANP’ are combined.

* *p*‐value is calculated using chi‐square statistics and *α* = 0.01. Compares answers provided by CNAs with answers provided by RNs regarding the interventions taken after observing signs and symptoms from the diagnostic strategy.

CNAs significantly more often communicate information on dehydration signs and symptoms to a (colleague) RN (*p *< 0.001). The interventions ‘I start a drink record chart’, ‘I perform additional physical examinations’ and ‘I give extra fluid through a hypodermoclysis’ were significant more frequently chosen by RNs than by CNAs. Some specific interventions, such as contacting the dietician and the speech therapist, were not included in this table as this answer option was only provided for one or two specific signs. When a resident was drinking less than normal, 11.6% of the RNs indicated to contact the dietician, whereas 15.5% of the CNAs did. Furthermore, when a resident had swallowing problems 66.4% RNs and 55.3% CNAs consulted the speech therapist.

## 4 DISCUSSION

The difficulty of identifying (a risk of) dehydration in nursing home residents is recognized internationally and requires knowledge of nursing staff (Bunn et al., 2018; Paulis et al., 2020). When asking Dutch registered nurses and CNAs using an open text question which signs and symptoms in a resident makes them think of dehydration, ‘confusion’, ‘change in urine colour’, ‘urinating less than normal’, ‘being drowsy’ and ‘decreased skin turgor’ were mentioned most often. When asking them the same question but including a pre‐structured list of signs and symptoms based on a diagnostic strategy to diagnose dehydration (Paulis et al., 2020), the anamnestic items ‘drinking less than normal’, ‘diarrhea’, ‘urinating less than normal’, ‘medication use related to dehydration’ and ‘fever’, were mentioned most often, as well as the physical symptoms ‘dry mucosa’, ‘change in urine colour’ and ‘dry incontinence material’. As ‘drinking less than normal’ is one of the main causes of dehydration (Hooper et al., 2015), it is striking that this was rarely mentioned (<5%) as a logical primary sign that made professionals think of dehydration in the open text question. However, when the sign ‘drinking less than normal’ was provided as an option in the pre‐structured list of signs and symptoms, it was most frequently chosen as being a contributor to dehydration. An explanation why RNs and CNAs might have not indicated ‘drinking less than normal’ as a first sign of dehydration in the open text question, could be that it is usually a combination of signs that trigger caregivers to think of dehydration, for instance the combination of ‘drinking less than normal’ and ‘diarrhea’ (Paulis et al., 2020). This is supported by previous research, indicating that single signs and symptoms lack diagnostic accuracy in older adults compared with a combination of signs and symptoms (Hooper et al., 2015; Paulis et al., 2020).

International studies have also shown that residents do not always reach the daily recommended fluid intake, which is 1,600–2,000 ml (Namasivayam‐MacDonald et al., 2014; Volkert et al., 2019). Dutch guidelines recommend 1,700 ml as daily fluid intake for older adults. In our study, the median fluid intake per day indicated by the RNs and CNAs was 1,400 ml, which is not in accordance with recommendations from the international literature and the Dutch guidelines. As a consequence, the question arises if nursing staff, as well as informal caregivers who take care of their family members, have sufficient knowledge to critically reflect on a sign such as ‘drinking less than normal’.

Our study also showed that 81% of the RNs and 75% of the CNAs did not receive any training on dehydration after they finished their initial professional education, while most of the them (36%) had >20 years working experience. Moreover, it seems that RNs and CNAs who received (post‐vocational) training on dehydration were more often triggered by signs and symptoms of the diagnostic strategy compared with RNs and CNAs who did not receive this post‐vocational training. Therefore, it seems to be important for nursing homes to make a plan on how and when to provide education on dehydration to their nursing staff and how often such education should be repeated. It seems also important to increase knowledge on dehydration among informal caregivers, for example by providing information brochures.

When looking at the signs and symptoms which did not made RNs and CNAs think of dehydration, we can see some differences between the two professional groups. The anamnestic items ‘vomiting’, swallowing problems’ and ‘presence of active diseases and the physical symptoms ‘higher pulse rate than normal’, ‘lower blood pressure than normal’ and ‘dry mucosa’ were less often indicated as factors to think of dehydration by CNAs compared with RNs. These differences might be due to differences in educational level. Within the professional education for RNs, more attention is paid to advanced clinical reasoning. This, together with their observational skills and knowledge of geriatric medicine, might give RNs a better basis to make proper clinical judgements compared with their CNA colleagues (Backhaus et al., 2015; Earleywine, 2011; Forsberg et al., 2011).

Participants indicated to observe the signs and symptoms related to dehydration very often themselves (92.6% of CNAs and 80.1% of RNs). CNAs significantly more often observes dehydration signs and symptoms themselves compared with RNs. The difference in the two groups may be explained by the fact that in Dutch nursing homes, RNs are less involved in regular caring tasks and more in acute and complex care situations (Backhaus, 2017). Besides direct patient related tasks, they also have management duties and execute a coaching role for colleagues with a lower educational level (Backhaus, 2017). Both CNAs and RNs also indicated to rely on other people for information quite often, such as informal caregivers (28.0% of CNAs and 29.5% of RNs). This could be explained by the fact that in Dutch nursing homes a variety professionals (nursing staff, medical staff, speech therapists, nutritional assistants, etc.) provide care to residents (Backhaus et al., 2016; Roberts & Ishler, 2018). Besides formal caregivers, informal care givers also often continue with their caring role after their relative is admitted to a nursing home, e.g., by providing assistance during mealtimes and during resident visits (Durkin et al., 2014; Roberts & Ishler, 2018). This enables the informal caregiver to observe, and report changes in the general condition of their relative (Powell et al., 2018). However, the fact that a large variety of caregivers are involved, emphasizes the importance of team‐based working in the nursing home. Furthermore, it leads to the question to what extent clear agreements and descriptions of roles and responsibilities are available. The involvement of multiple formal and informal caregivers could lead to unclear agreements on who is responsible for observing changes in residents, and therefore to ineffective observation of dehydration signs and symptoms. Therefore, it is essential to have clear agreements among nursing home staff (incl. nutrition assistants) and informal caregivers about a shared responsibility in this (Baik, 2017).

Most actions taken by RNs and CNAs after observing a sign or symptom related to dehydration can be found in communicating this finding to other care professionals in the nursing home (NHP, ANP, to colleague RNs or CNAs or to the care coordinator). What we can conclude, based on the results of our study, is that nursing staff is involved in the presumption phase of the diagnostic strategy, and provides information about the presence of signs and symptoms in this phase to colleague NHPs or ANPs. This information can provide a basis to start the confirmation phase (including blood tests). Nevertheless, the results of this study also show that besides RNs and CNAs, various other care providers play a role in this presumption phase. Therefore, more attention should be paid to the role other care professionals and the informal caregiver have in this presumption phase. By clearly describing which professionals are responsible for the presumption phase and who they should communicate their findings to, there might be a better recognition of signs and symptoms related to dehydration among residents, and more focus on prevention and its treatment.

### 4.1 Limitations

To the authors’ best knowledge, this is the first study investigating the awareness among RNs and CNAs about signs and symptoms among residents related to dehydration, and the interventions taken when these signs and symptoms are observed. Therefore, the results of this study are a valuable contribution to dehydration research in the nursing home setting. However, some limitations should be mentioned. In our study, 250 RNs and 226 CNAs participated. Due to the anonymity of the survey responses, we do not know if participants are equally distributed across the country, which may affect the generalizability of the results. Another limitation of this study is that the diagnostic dehydration strategy is based on a literature review and on expert opinion. This expert panel consisted of NHPs and ANPs, meaning there were no RNs or CNAs involved. If RNs and CNA would have been involved, different anamnestic items or physical symptoms related to dehydration might have been included in the diagnostic strategy. However, as the diagnostic strategy is also based on all available literature in the field, we believe the consequences of this choice will be limited. Not all questions had the exact same answer categories: for the question which interventions RNs and CNAs take when they observe a dehydration sign or symptom in a resident, ‘contacting the dietician’ or ‘contacting the speech therapist’ could only be chosen as an answer option for the items ‘drinking less than normal’ and ‘swallowing problems’. This could have affected the answer option ‘other’. Lastly, residents were not involved in this study as the main focus of this study was examining the identification of dehydration and interventions taken by RNs and CNAs in the nursing home. Even though, the RNs and CNAs in Dutch nursing homes interact intensively with residents, it is preferable to include residents in future research to make proper improvements in dehydration care on client level with a focus on shared decision‐making and team‐based care.

## 5 CONCLUSION

Registered nurses and CNAs working in Dutch nursing homes recognize most of the signs and symptoms from an international dehydration strategy as factors associated with dehydration. Furthermore, CNAs and RNs indicated that they mostly observe these signs and symptoms of dehydration themselves, but that they also receive information on the presence of these signs and symptoms in residents from colleagues and/or informal caregivers. Actions most frequently taken by participants after observing the factors associated with dehydration is informing the nursing home physician or the advanced nurse practitioner. The fact that professionals in the nursing home often rely on information about dehydration signs and symptoms from colleagues or informal caregivers strengthens the need for team‐based working and makes it essential that there is a clear description of roles and responsibilities on dehydration care. Without this, it is likely that (a risk of) dehydration is left unattended. Furthermore, it appeared that CNAs and RNs often did not receive any dehydration training after entering workforce. Therefore, it could be less likely they recognize signs and symptoms from the diagnostic strategy as a sign for dehydration. As a consequence, it is recommended to structurally organize dehydration training in nursing homes. This may lead to better recognition of signs and symptoms related to dehydration among residents, and more focus on prevention and its treatment.

## CONFLICT OF INTEREST

All authors declare that there has been no conflict of interest.

## AUTHOR CONTRIBUTIONS

All authors met the four criteria for authorship: All authors have (1) made substantial contributions to conception and design, or acquisition of data, or analysis and interpretation of data; (2) have been involved in drafting the manuscript or revising it critically for important intellectual content; (3) have given final approval of the version to be published. Each author should have participated sufficiently in the work to take public responsibility for appropriate portions of the content; and (4) have agreed to be accountable for all aspects of the work in ensuring that questions related to the accuracy or integrity of any part of the work are appropriately investigated and resolved.

### PEER REVIEW

The peer review history for this article is available at https://publons.com/publon/10.1111/jan.15032.

## Supporting information

Supplementary Material

Supplementary Material

## Data Availability

Data available on request from the authors.
