# Supplementary material for: Dehydration in the nursing home: Recognition and interventions taken by Dutch nursing staff
Source: J Adv Nurs. 2021 Aug 30;78(4):1044–54. doi: 10.1111/jan.15032 (PMC9290809; doi:10.1111/jan.15032)
Supplement: Supplementary file 1 — Supplementary Material [file JAN-78-1044-s001.doc]

Additional file 1 Questionnaire

**Detecting dehydration, how do we do that?**

- **Can we use your answers (anonymously) for scientific research?**
- Yes
- No
- **Where do you work?**
- In the nursing home
- Other

**GENERAL QUESTIONS**

1. **What is your profession?**

- Certified nurse assistant
- Registered nurse

1. **How many years of working experience in the nursing home you have?**

- 0-5 years
- 5-10 year
- 10-15 years
- 15-20 years
- >20 years

1. **Do you (mainly) work with somatic or psychogeriatric patients?**

- (mainly) somatic patients
- (mainly) psychogeriatric patient
- both

1. **Did you receive training on dehydration during your education?**

- Yes
- No

1. **Did you receive training on dehydration after your education?**

- Yes
- No

1. **Is there a protocol/guideline for diagnosing and treating dehydration in the nursing home you work at?**

- Yes
- No
- I don`t know

1. **Which minimum amount of fluid per day per resident is pursued in your nursing home? (regardless of wheather conditions etc.)**

**
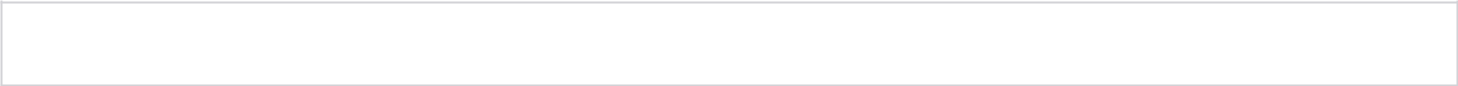
**

1. **Which signs/symptoms the resident displays trigger you to think of dehydration?**

**
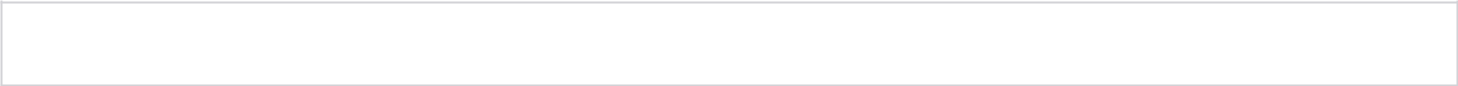
**

**SYMPTOMATOLOGY**

In daily practice you probably see nursing home residents who are at risk to develop dehydration or may already be dehydrated. We will now list a number of signs and symptoms that can occur in nursing home residents with (a risk of) dehydration. We would like to ask you to consider the following questions:

- Do you think of dehydration when this sign/symptom is present?
- Do you signal the sign/symptom yourself or do you receive information from someone else?
- What action do you take when the sign/symptom is present in a resident?

Note: There are no good or false answers. We only want to gain insight in how dehydration care is being performed in the nursing home you work in.

Note: Below you can see the structure of the questionnaire for the sign/symptom ‘vomiting’. The structure of the questionnaire looks exactly the same for the signs/symptoms: diarrhea, urinating less than normal, presence of active disease(s) (e.g., infection), medication use related to dehydration (e.g., diuretics, lithium and anticholinergic meds), fever, dry incontinence material, change in behaviour, lower blood pressure than normal, dry mucosa, rapid weight loss (>1kg/day), dry longitudinal furrowed tongue, higher pulse rate than normal and change in urine colour. For ‘drinking less than normal’, an extra answer option was added namely ‘contacting the dietician’. In addition, ‘swallowing problems’ contains the extra answer option ‘contacting the speech therapist’.

**‘Vomiting’**

- **When a resident is vomiting, do you think of (a risk of) dehydration?**
- Always
- Sometimes
- Never thought this could be a sign of dehydration
- **Do you usually notice yourself that a resident is vomiting, or do you hear this from someone else? (more options are possible)**
- I notice this myself
- The nurse assistant notices this and informs me about it
- The certified nurse assistant notices this and informs me about it
- The care coordinator notices this and informs me about it
- The registered nurse notices this and informs me about it
- The nursing home physician or advanced nurse practitioner notices this and informs me about it
- The informal caregiver notices this and informs me about it
- This sign/symptom is not being noticed at all
- **When you notice that a resident is vomiting, what do you do? (more options are possible)**
- I write it down in the agenda for the nursing home physician/ advanced nurse practitioner visit
- I communicate this to the nursing home physician / or advanced nurse practitioner
- I communicate this to a (colleague) registered nurse
- I communicate this to the care coordinator
- I communicate this to a (colleague) certified nurse assistant
- I communicate this to a (colleague) nurse assistant
- I start a drink record chart
- I give the resident more fluid
- I give extra fluid through a hypodermoclysis
- I perform additional physical examinations
- I request blood tests
- I don`t do anything
- Other ….. (fill in)


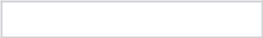


**Last Question: Can we approach you for participation in follow-up research? If yes, please write down your e-mail address**

- Yes
- No

**Thank you for your participation!**

Click further to save your answers!
