# Supplementary material for: Dehydration in the nursing home: Recognition and interventions taken by Dutch nursing staff
Source: J Adv Nurs. 2021 Aug 30;78(4):1044–54. doi: 10.1111/jan.15032 (PMC9290809; doi:10.1111/jan.15032)
Supplement: Supplementary file 2 — Supplementary Material [file JAN-78-1044-s002.docx]

Additional file 2 Other caregivers providing information about signs and symptoms related to dehydration to CNAs and RNs

|  | **CNA (n=226)†** | | | | | | | **RN (n=250)‡** | | | | | | |
| --- | --- | --- | --- | --- | --- | --- | --- | --- | --- | --- | --- | --- | --- | --- |
|  | **NA (%)§,**†† | **CNA (%)†,**†† | **Care coordinator (%)**†† | **RN (%)‡,**†† | **NHP/ANP (%)¶,**†† | **Informal caregiver (%)**†† | **Not observed (%)**†† | **NA (%)§,**†† | **CNA (%)†,**†† | **Care coordinator (%)**†† | **RN (%)‡,**†† | **NHP/ANP (%)¶,**†† | **Informal caregiver (%)**†† | **Not observed (%)**†† |
|  |  |  |  |  |  |  |  |  |  |  |  |  |  |  |
| **Drinking less than normal** | 48.2% | 48.7% | 20.8% | 23.0% | 13.7% | 43.4% | 2.2% | 58.0% | 70.8% | 26.8% | 26.8% | 13.6% | 49.6% | 3.6% |
| **Vomiting** | 53.1% | 56.2% | 23.0% | 31.0% | 5.8% | 48.2% | 0.4% | 68.0% | 83.2% | 32.8% | 33.6% | 6.8% | 51.6% | 2.0% |
| **Diarrhea** | 59.3% | 61.5% | 28.8% | 30.1% | 5.3% | 36.7% | 0.4% | 71.6% | 84.8% | 36.4% | 36.8% | 7.6% | 43.2% | 2.0% |
| **Urinating less than normal** | 57.1% | 61.5% | 27.4% | 31.0% | 4.4% | 28.8% | 3.1% | 63.6% | 82.0% | 36.0% | 33.6% | 5.6% | 31.6% | 4.4% |
| **Medication use related to dehydration** | 27.4% | 50.9% | 31.9% | 43.4% | 31.4% | 17.3% | 4.9% | 25.6% | 53.6% | 31.2% | 34.4% | 37.6% | 15.2% | 5.6% |
| **Presence of active disease(s)** | 50.4% | 59.7% | 32.7% | 38.9% | 28.3% | 28.3% | 1.3% | 50.4% | 78.0% | 40.8% | 38.0% | 28.0% | 30.8% | 2.4% |
| **Change in behaviour** | 59.3% | 61.1% | 30.5% | 33.6% | 13.3% | 46.0% | 1.3% | 64.4% | 83.6% | 38.8% | 36.0% | 10.4% | 49.2% | 3.2% |
| **Swallowing problems** | 55.3% | 59.3% | 31.4% | 32.7% | 11.9% | 40.3% | 1.8% | 61.2% | 80.4% | 36.4% | 34.8% | 13.2% | 42.0% | 3.6% |
| **Fever** | 51.8% | 65.5% | 33.6% | 39.4% | 16.8% | 29.6% | 0.4% | 54.4% | 83.6% | 41.2% | 38.4% | 14.8% | 30.4% | 1.6% |
| **Lower blood pressure than normal** | 17.3% | 58.8% | 32.3% | 37.6% | 24.3% | 7.1% | 4.9% | 18.4% | 74.0% | 36.8% | 37.6% | 19.2% | 6.8% | 5.2% |
| **Dry mucosa** | 54.4% | 60.6% | 32.3% | 37.2% | 12.8% | 30.5% | 1.3% | 54.4% | 78.8% | 36.4% | 35.6% | 14.8% | 30.8% | 3.6% |
| **Rapid weight loss** | 45.6% | 59.7% | 31.4% | 35.0% | 11.9% | 20.8% | 5.3% | 49.6% | 82.0% | 38.8% | 37.6% | 14.8% | 14.8% | 7.2% |
| **Dry longitudinal furrowed tongue** | 47.3% | 56.6% | 29.6% | 34.5% | 15.0% | 26.5% | 7.5% | 44.4% | 72.0% | 34.0% | 33.6% | 20.4% | 21.6% | 13.2% |
| **Higher pulse rate than normal** | 22.6% | 58.0% | 31.0% | 38.5% | 21.2% | 6.6% | 6.2% | 20.8% | 75.6% | 36.0% | 38.4% | 19.2% | 9.2% | 6.0% |
| **Change in urine colour** | 60.6% | 62.4% | 34.1% | 34.5% | 6.2% | 22.6% | 0.9% | 62.0% | 85.2% | 39.6% | 37.6% | 6.8% | 23.6% | 2.0% |
| **Dry incontinence material** | 60.6% | 62.8% | 33.6% | 32.7% | 3.5% | 15.9% | 0.0% | 63.6% | 84.4% | 38.4% | 35.2% | 6.8% | 21.6% | 4.0% |
|  | ***Mean 48.2%*** | ***Mean 59.0%*** | ***Mean 30.3%*** | ***Mean 34.6%*** | ***Mean 14.1%*** | ***Mean***  ***28.0%*** | ***Mean***  ***2.6%*** | ***Mean 51.9%*** | ***Mean 78.3%*** | ***Mean 36.3%*** | ***Mean 35.5%*** | ***Mean***  ***15.0%*** | ***Mean 29.5%*** | ***Mean 4.4%*** |

† CNA = Certified Nurse Assistant

‡ RN = Registered Nurse

§ NA = Nurse Assistant

¶ NHP/ANP = Nursing Home Physician / Advanced Nurse Practitioner

†† Percentages are calculated on the answers given by participants (CNAs or RNs) how often each group of caregivers reports on the presence of a sign/symptom related to dehydration
